# Effect of graphite oxide and exfoliated graphite oxide as a modifier for the voltametric determination of dopamine in presence of uric acid and folic acid

**DOI:** 10.1038/s41598-021-01328-w

**Published:** 2021-12-15

**Authors:** H. Vidya, B. E. Kumara Swamy, S. C. Sharma, G. K. Jayaprakash, S. A. Hariprasad

**Affiliations:** 1 Department of P. G. Studies and Research in Industrial Chemistry, Kuvempu University, Jnanasahyadri, Shankaraghatta, Shimoga, Karnataka 577 451 India; 2 National Assessment and Accreditation Council, Jain University, Bangalore, Karnataka 560 069 India; 3 School of Energy Science and Engineering, Indian Institute of Technology Guwahati, Guwahati, 781039 India; 4 School of Advanced Chemical Sciences, Shoolini University, Bajhol, Himachal Pradesh 173229 India; 5 Jain University, Bangalore, Karnataka 560 069 India

**Keywords:** Biochemistry, Chemistry, Materials science, Nanoscience and technology

## Abstract

In the present work, exfoliated graphite oxide (E-GO) was prepared by sonicating graphite oxide (GO) (prepared by modified Hummer’s and Offemam methods). Prepared GO and E-GO were characterized using infrared absorption spectroscopy, X-ray diffraction, and scanning electron microscopy. The electrocatalytic properties of GO and E-GO towards detection of dopamine (DA), uric acid (UA), and folic acid (FA) were investigated using cyclic voltammetry and differential pulse voltammetry. Our results revealed that E-GO has a slighter advantage over the GO as an electrode modifier for detection DA, UA, and FA, which might be ascribed to the good conductivity of E-GO when compared to the GO.

## Introduction

Graphite oxide, also known as graphitic oxide or graphitic acid, is a variable-ratio carbon, oxygen, and hydrogen compound produced by treating graphite with powerful oxidizers and acids to remove excess metals^1–5^. At the GO surface, carbon atoms are embedded along with oxygen-containing functional groups such as keto and epoxy groups across the basal plane, the phenolic and carboxyl groups at the edges. In general GO conductivity is poor therefore modified GO surfaces like exfoliated graphite oxide (E-GO) are preferred for electrochemical sensing applications^6–12^. E-GO has strong hydrophilicity and electrostatic repulsion effects therefore, carboxylic acid groups attached to GO could ionize in water, resulting in minor GO sheets have carboxylic ions containing negative charges. In addition to this E-GO has lesser thickness when compared to GO. Which makes it exhibit good electroanalytical performance better than other carbon-based nanomaterials^13,14^. Banks et al. recently stated that an improved electron transmission of graphene takes place on its edges and that the presence of oxygen groups on its edges can affect molecular adsorption/desorption before and after an electrochemical reaction^14^. Therefore GO is one of the preferred carbon materials for electrochemical applications. In general E-GO conductivity is higher than the GO, therefore it will be very interesting to compare the electrocatalytic activities of E-GO over GO for sensing applications.

DA is an important neurotransmitter in the mammalian central nervous system and its detection has attracted much interest because a change in DA levels is linked with the understanding of brain functions^15^. Methods for the detection of DA include Chemiluminescence^16^, Fluorimetry^17^, Capillary Electrophoresis^18^, and Voltammetry^19^. Among these methods, voltammetric methods are popular due to their low cost and user-friendly nature.

Uric acid (2,6,8–trihydroxypurine, UA), is also an important biomolecule present in the human body with the DA and its abnormal levels lead to many clinical disorders (gout, kidney, and cardiac problems)^20,21^. Therefore, exploring a reliable method for the determination of uric acid is also interesting. Electrochemical methods are useful for detecting DA and UA simultaneously^22–31^.

Folic acid (FA) and its derivatives are required in the single-carbon metabolism process such as amino acid and nucleic acid biosynthesis^32^ as well as in cell division and growth. This vitamin is essential for rapid cell growth like blood production, especially during pregnancy. The deficiency of FA can cause health problems^33,34^. Methods like chromatography^35,36^, flow-injection chemiluminometry^37^, and Spectrophotometry^38^ can be used to detect FA. However, when compared to these methods voltammetric methods are more popular for FA determination because of their convenience. Simultaneous determination of DA, UA, and FA is very important and useful as explained by Abdelwahab and Shim^39^.

To enhance the selectivity and sensitivity of carbon paste electrodes (CPE) different modifiers have been used^40–43^. GO and E-GO modified electrodes have been used to detect redox moieties like lead^10^, L-dopa^8^, and acetaminophen^13^. As for our best knowledge till now no reports published on E-GO modified CPE for the simultaneous detection of DA, UA, and FA. In this study, CPE is modified by E-GO for sensing DA, FA, and UA as part of the studies we have compared results with GO-modified CPE.

## Experimental

### Materials and stock solution

**Scheme 1:**
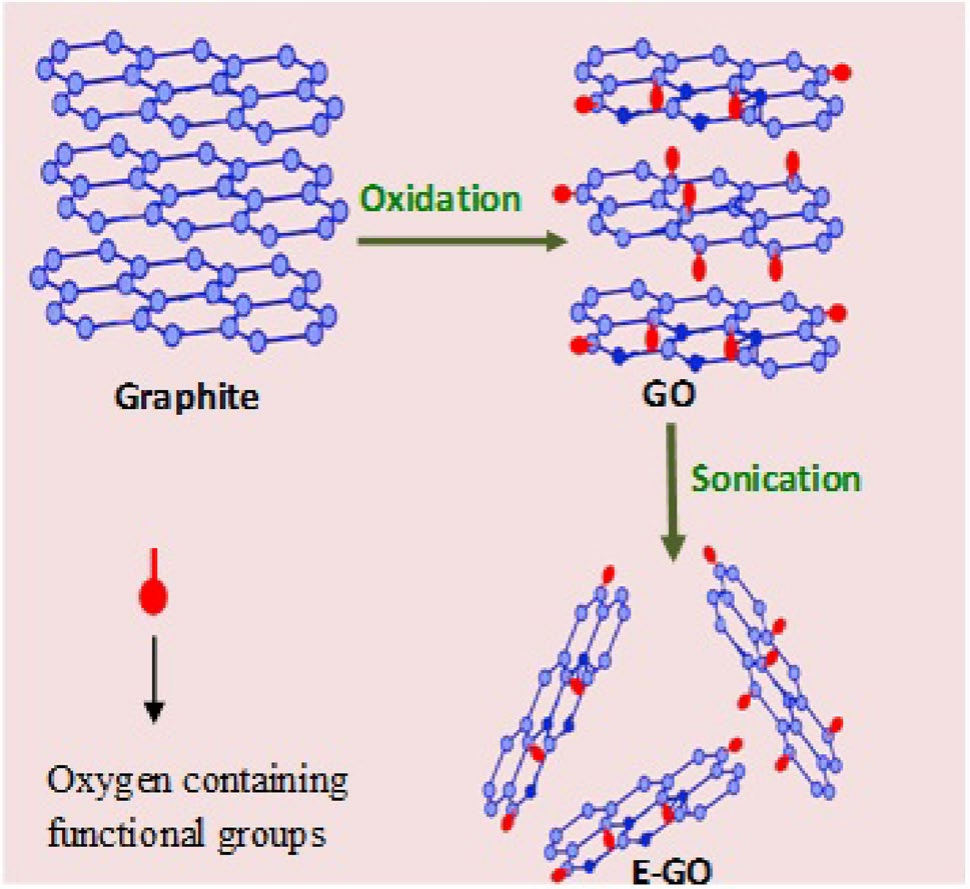
A feasible mechanism of synthesis of GO and E-GO.

DA, UA, FA, disodium hydrogen phosphate (Na_2_HPO_4_), sodium dihydrogen orthophosphate (NaH_2_PO_4_), and silicon oil were purchased from Himedia chemicals. Sodium hydroxide (NaOH), perchloric acid (HClO_4_), potassium chloride (KCl), and graphite powder were purchased from Merck chemical. Stock solutions of DA were prepared in 0.1 M perchloric acid, UA, and FA in 0.1 M NaOH respectively. All chemicals are of analytical grade quality and were used without further purification. The preparation of Graphite oxide (GO) was carried out using harsh oxidation using the Hummers method as described in previous literature^2^. Then GO powder was dispersed in double-distilled water and sonicated to exfoliate the Graphite oxide (E-GO). A feasible mechanism of synthesis of GO and E-GO is shown in Scheme 1.

### Apparatus

X-ray diffraction (XRD) patterns of Graphite and GO samples are obtained using PHILIPS PW3710 diffractometer (Cu, kα radiation) with a step scan at 0.2^0^. Fourier transformed infrared (FT-IR, Perkin-Elmer) spectra in KBr pellet were recorded to detect the functional group of GO and E-GO. Field emission scanning electron microscope (FE-SEM-supra 40VP, Carl Zees) were also obtained for the Graphite, GO and E-GO are used for knowing microstructure and energy dispersive X-ray (EDX) analysis are also measured for elemental composition of E-GO.

All electrochemical experiments were performed using CH instrument model 660c. The electrode system comprises a bare or modified CPE (GO/E-GO) as a working electrode. Potentials were measured using an auxiliary electrode and saturated calomel as a reference electrode. All voltammetric curves were recorded at room temperature and phosphate buffer solution (PBS) pH = 7.0 is used as an electrolyte.

### Preparation of bare CPE and modified CPE

The bare CPE was prepared by grinding a 70:30 ratio of graphite powder and silicone oil in an agate mortar. The pestle was used to obtain a homogeneous paste. A portion of the resulting homogeneous paste was packed into the cavity of a Teflon tube and polished using smooth paper. Bare CPE was modified by adding (0, 2, 4, 6, 8 & 10) mg GO/E-GO to the above-mentioned graphite powder and silicon oil mixture.

## Results and discussion

### Characterization

**Figure 1:**
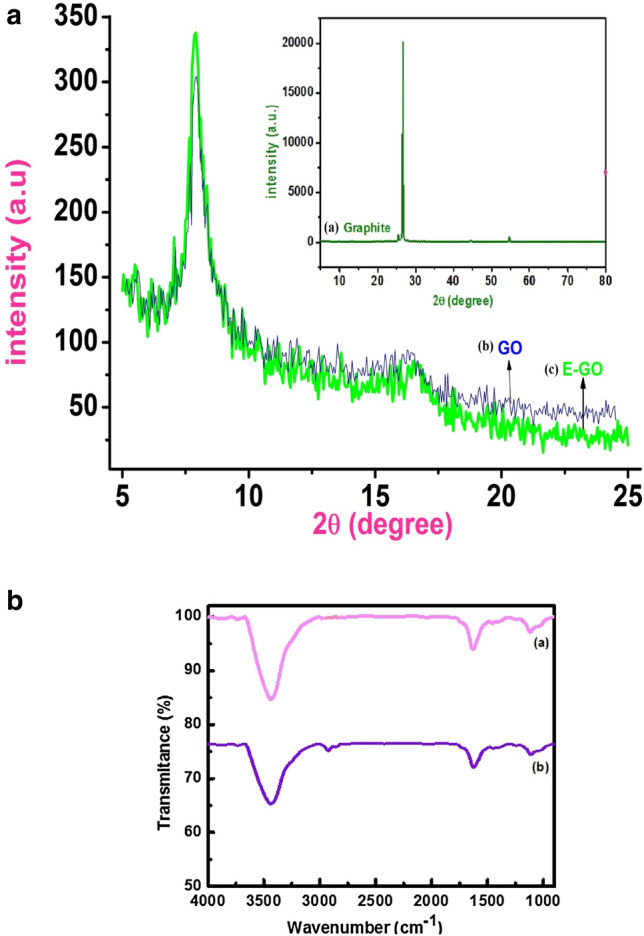
(**a**) Powder XRD pattern of Graphite and Graphite oxide (inset). (**b**) FT-IR spectra of (a) GO and (b) E-GO.

Figure 1a shows a typical XRD pattern for Graphite and Graphite oxide (inset). The interlayer distance changes after the oxidation of graphite and it was calculated based on 2θ position by using Bragg’s law. During the oxidation process, the functional groups such as carboxyl group and epoxy attached to the precursor graphite, these interlayer spacing of Graphite shift 3.5 A^0^ to 11.2 A^0^ (GO). The small inter-layer spacing of graphite suggests a dense packing when compared to the GO, but the peak intensity for GO was reduced significantly. These results indicate that most of the GO layer exfoliated randomly while a small position of the sample remained as a layered structure. But the sonication effect leads to exfoliates the more GO and this corroboration was shown in the FESEM image, discussed below.

The FT-IR spectra of (a) GO and (b) E-GO was ground with KBr and pelletized oxidation involves the content of oxygen-related functional groups shown in Fig. 1b. Broadband at 3432 cm^−1^ is attributed to the stretching frequency of O–H bond and the band at 1730 cm^−1^ is due to stretching of the C=O bond of carbonyl or carboxyl group situated at the edges of GO sheets. The peak at 1623 cm^−1^ is due to aromatic C=C bond and the peak at 1050 cm^−1^ is due to the epoxy (–O–) groups^44^.

**Figure 2:**
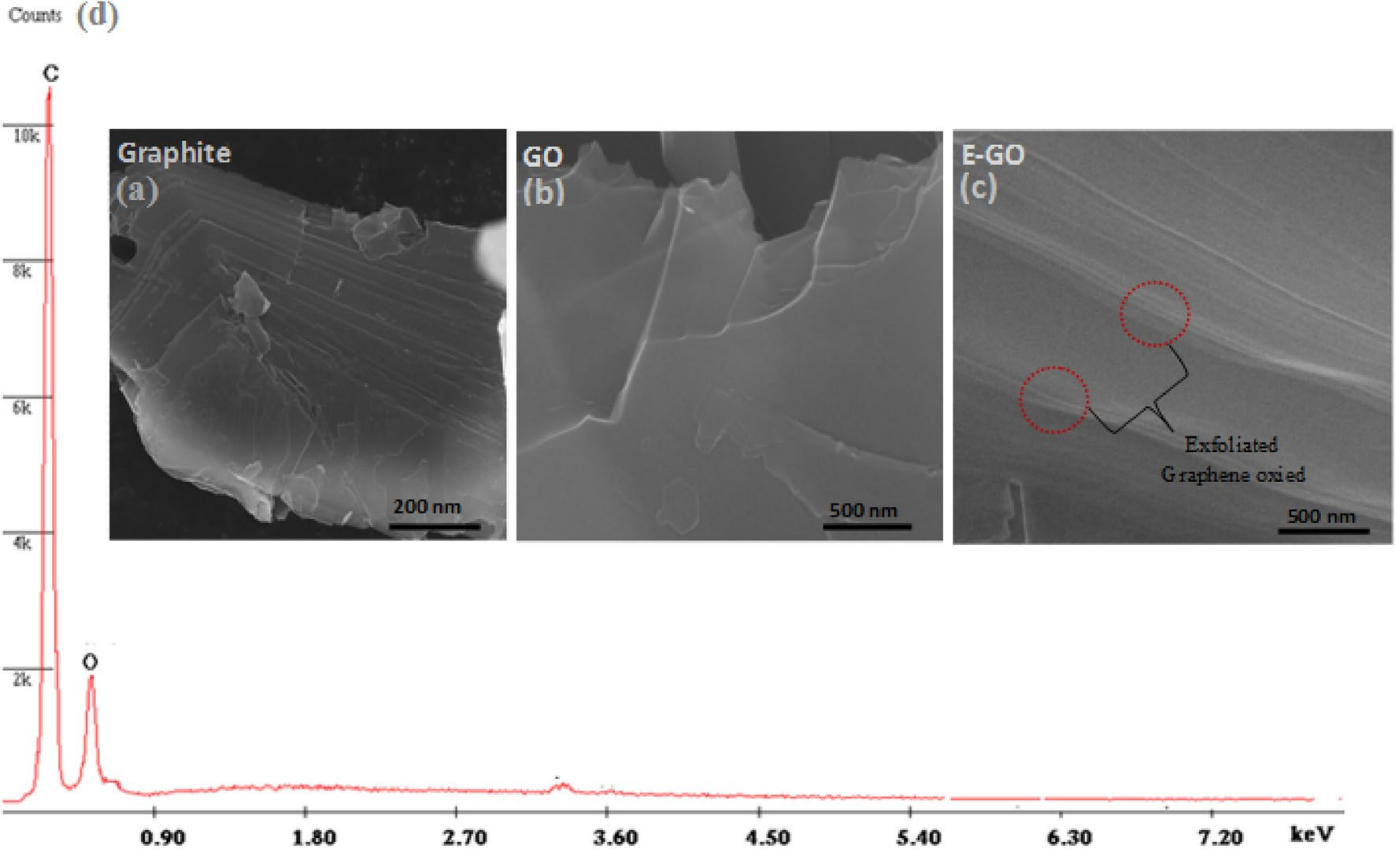
SEM image of the powdered (**a**) Graphite, (**b**) GO, and (**c**) E-GO sample and the EDX spectra.

The morphological changes upon sonication and exclusive of sonication are analyzed by FESEM. Figure 2a shows the image of graphite powder which demonstrated the flat surface and stacked structure and the graphite oxide exhibits random wrinkled layer structure seen in Fig. 2b**.** This indicates the ordered layer structure in pristine graphite has been disrupted due to oxidation. Upon sonication of graphite oxide for 2.5 h the GO exfoliate and the interlayer distance increased tremendously can be observed in Fig. 2c. The EDX spectra are shown in Fig. 2d C and O peaks and there are no other elemental impurities.

### Optimization of modifiers at bare CPE

**Figure 3:**
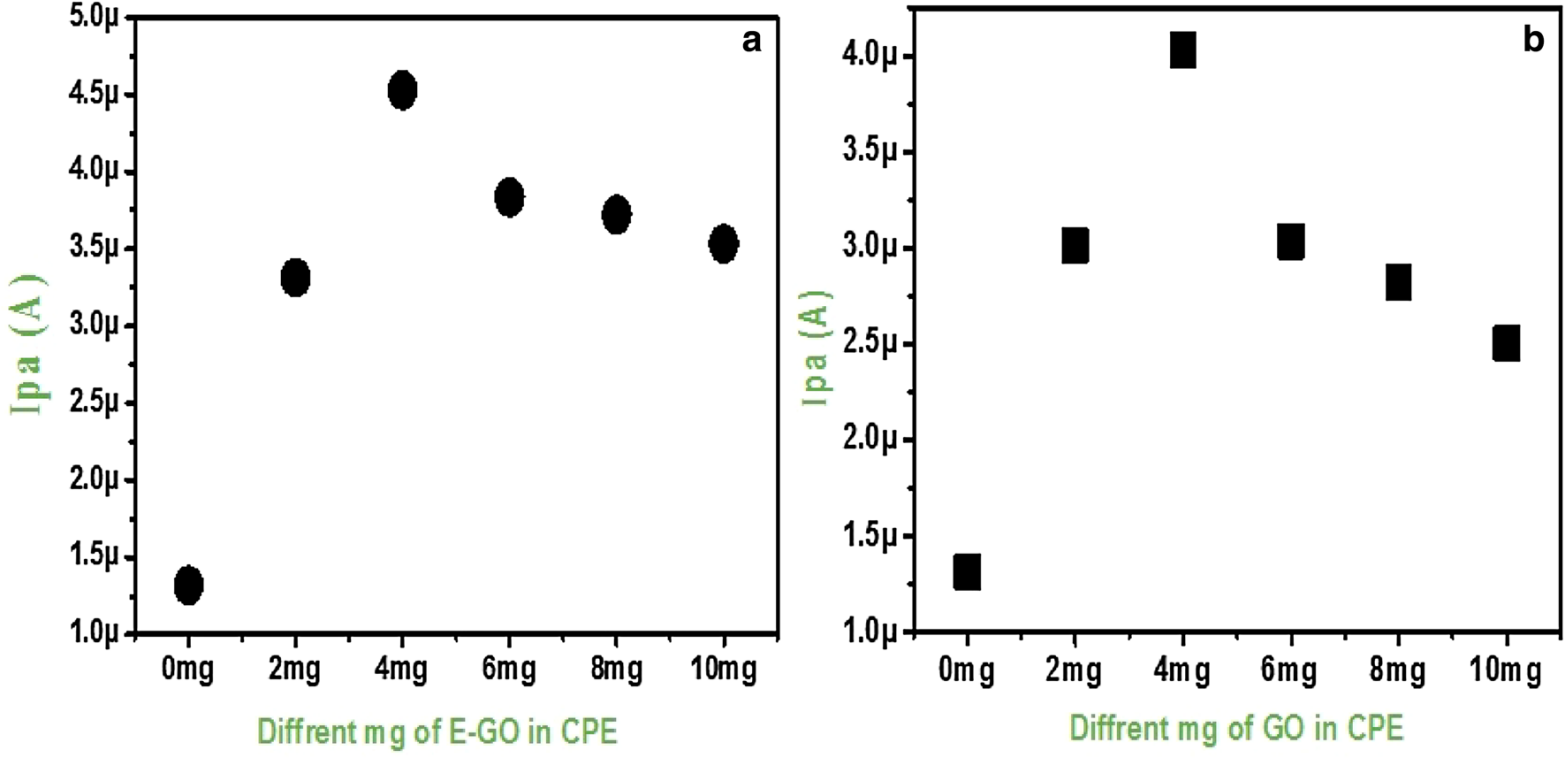
Graph of Ipa vs quantity of (**a**) GO and (**b**) E-GO in carbon paste electrode.

To investigate the effect of the concentration of modifiers (GO and E-GO) on the electrode response and to find the optimum amount some electrodes containing 0, 2, 4, 6, 8, and 10 mg were prepared. The cyclic voltammograms were recorded for both the (GO/E-GO) modified electrodes at a sweep rate of 50 mVs^−1^ under identical conditions. Figure 3a shows the plot of anodic peak current versus different amounts of the modifier. With increasing the amount of modifier anodic peak current increased and however, above 4 mg increasing the amount leads to undesirable properties, which correspondingly decreased the electron transfer rate. Figure 3b shows similar properties but the bare CPE enclose GO shows a slight decrease in the anodic peak current due to less exfoliation of GO. Consequently, for both the modifiers the electrode comprising 4 mg (GO and E-GO) in the bare CPE is more active and it was chosen as the optimum amount.

### Electrochemical response of DA, UA, and FA at bare CPE and modified CPE

**Figure 4:**
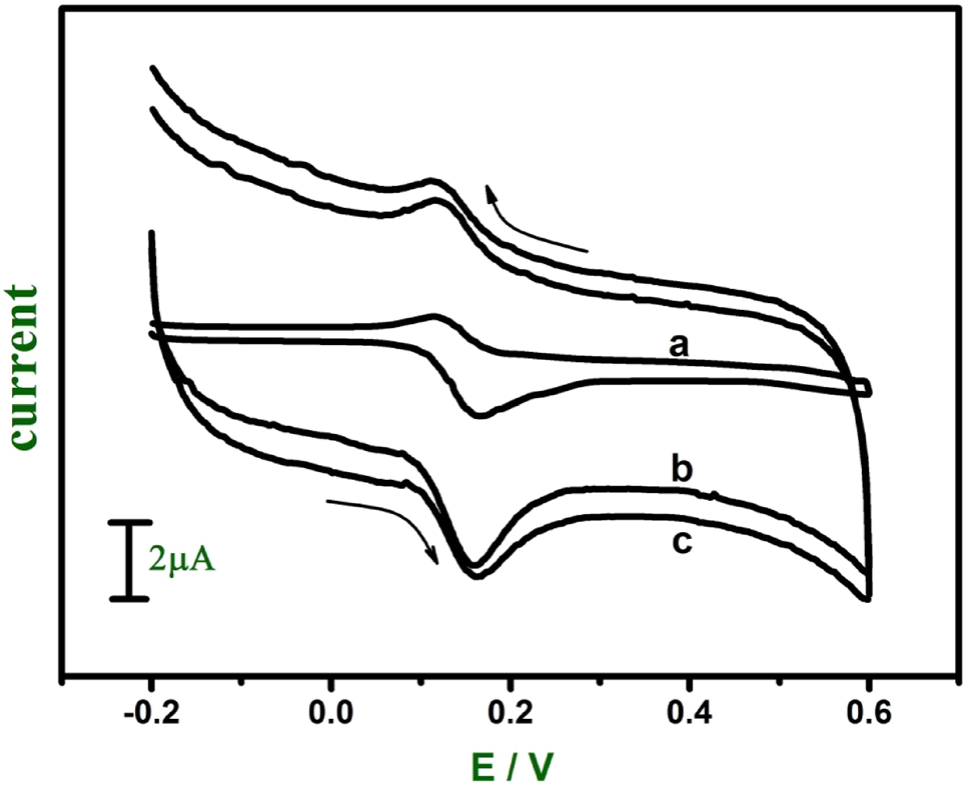
Cyclic voltammograms of 0.02 mM DA at the bare CPE (curve **a**), GO (curve **b**) and E-GO (curve **c**) in 0.2 M phosphate buffer solution at pH 7.0 with sweep rate 0.05 Vs^−1^.

**Scheme 2:**
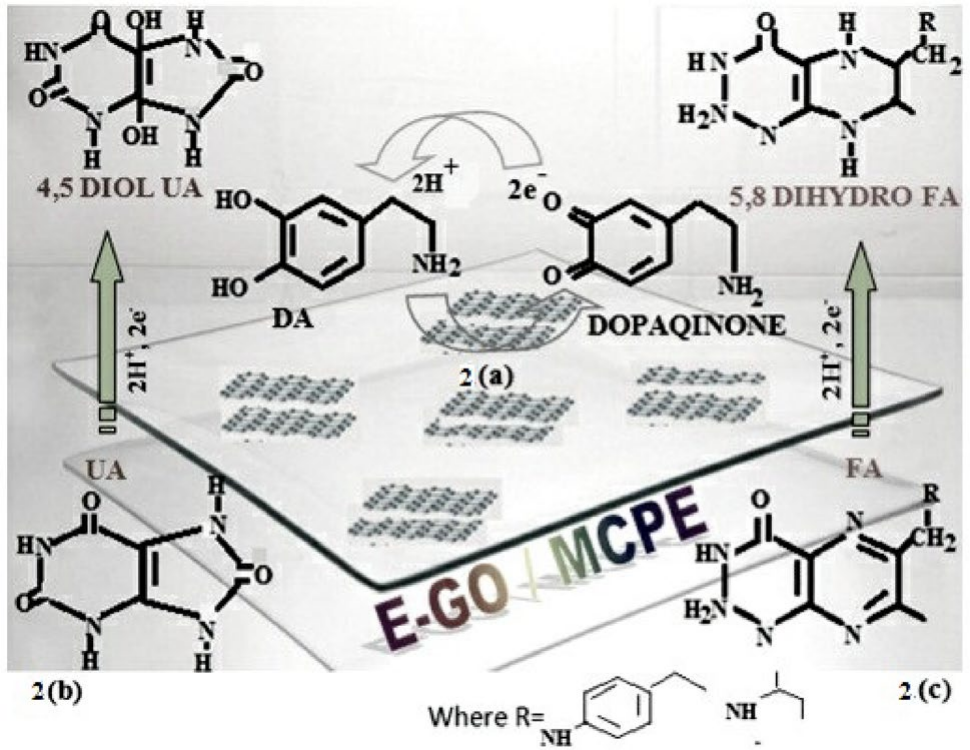
A feasible schematic illustration of the catalysis of (**a**) DA (**b**) UA (**c**) FA.

Figure 4 shows the CV of 0.02 mM DA at the bare CPE (curve a), GO modified CPE (curve b) and E-GO modified CPE (curve c) in 0.2 M phosphate buffer solution (PBS) at pH 7.0. The peak separation potential ΔEp = (Epa (anodic peak potential) − Epc (cathodic peak potential) ) for curves a, b, and c was found to be 49, 42, and 51 mV respectively which indicates the rate of reaction is fast and reversible electron transfer takes place. Under the same condition the anodic peak current for curves a, b and c was increased significantly. We can observe that for curve c there was an increase in anodic peak current compare to curve b due to the high conductivity of E-GO. Moreover, the DA molecules penetrate through the interlamellar space of negatively charged GO and E-GO^45^. The detailed oxidation mechanism of DA at modified electrodes was shown in Scheme 2a^4^.

**Figure 5:**
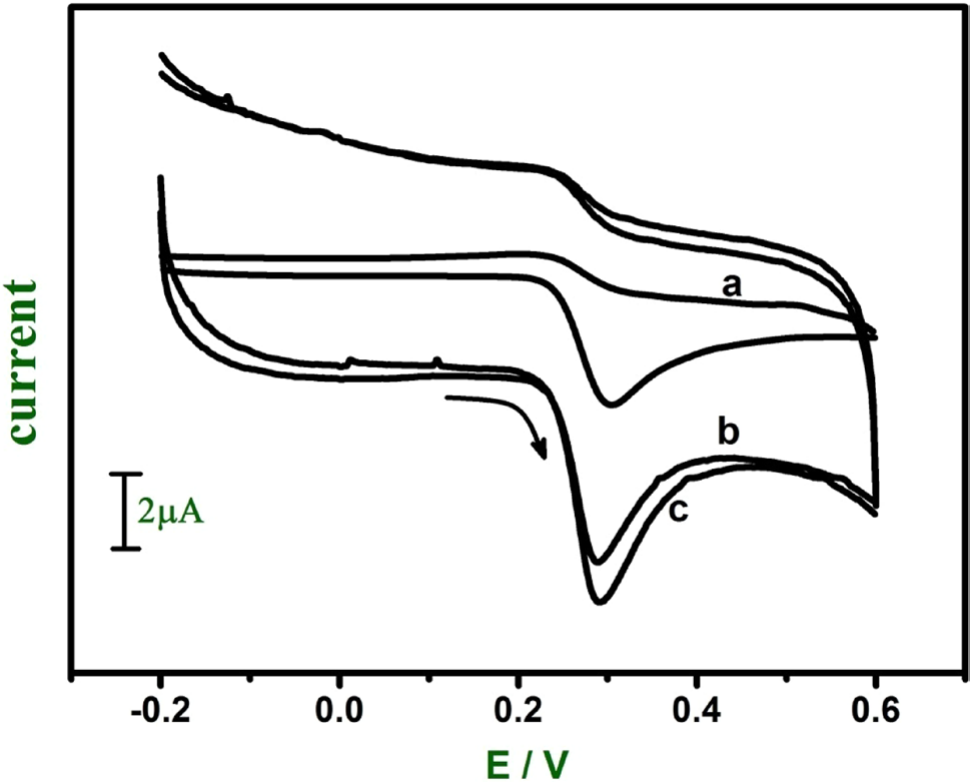
Cyclic voltammograms of 0.06 mM UA at the bare CPE (curve **a**), GO (curve **b**) and E-GO (curve **c**) in 0.2 M phosphate buffer solution at pH 7.0 with sweep rate 0.05 Vs^−1^.

**Figure 6:**
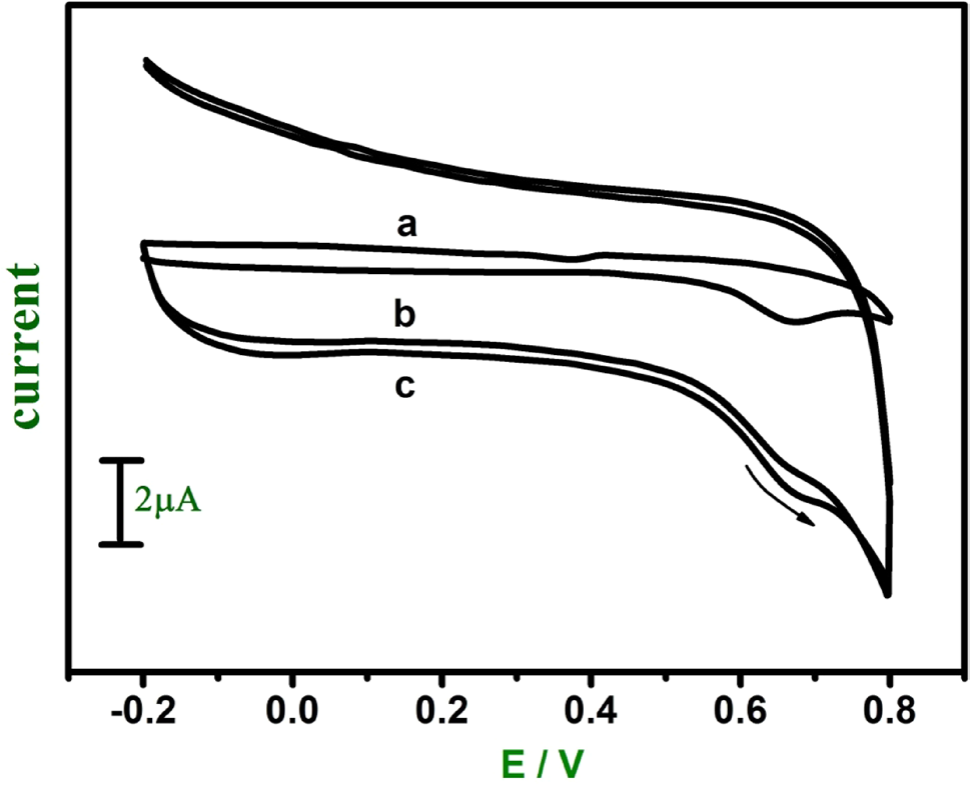
Cyclic voltammograms of 1 mM FA at the bare CPE (curve **a**), GO (curve **b**) and E-GO (curve **c**) in 0.2 M phosphate buffer solution at pH 7.0 with sweep rate 0.05 Vs^−1^.

The electrochemical response of UA (0.06 mM) and FA (1 mM) was studied in a 0.2 M phosphate buffer at pH 7.0. Both UA and FA show irreversible electron transfer at modified electrodes. Figure 5 shows the anodic peak potential of UA at bare CPE (curve a), GO modified CPE (curve b) and E-GO modified CPE (curve c) were observed at 302 mV, 287 mV, and 290 mV respectively. At both modified CPE anodic peak potential shifted towards the negative direction. Figure 6 shows the anodic peak potential of FA at bare CPE (curve a), GO modified CPE (curve b) and E-GO modified CPE (curve c) were observed at 671 mV, 665 mV, and 685 mV respectively and showed the increase in the electrocatalytic effect of the modified CPE is compared to bare CPE. The proposed oxidation mechanism of UA and FA at modified CPE was shown in Scheme 2b,c^46,47^.

### Effect of sweep rate

**Figure 7:**
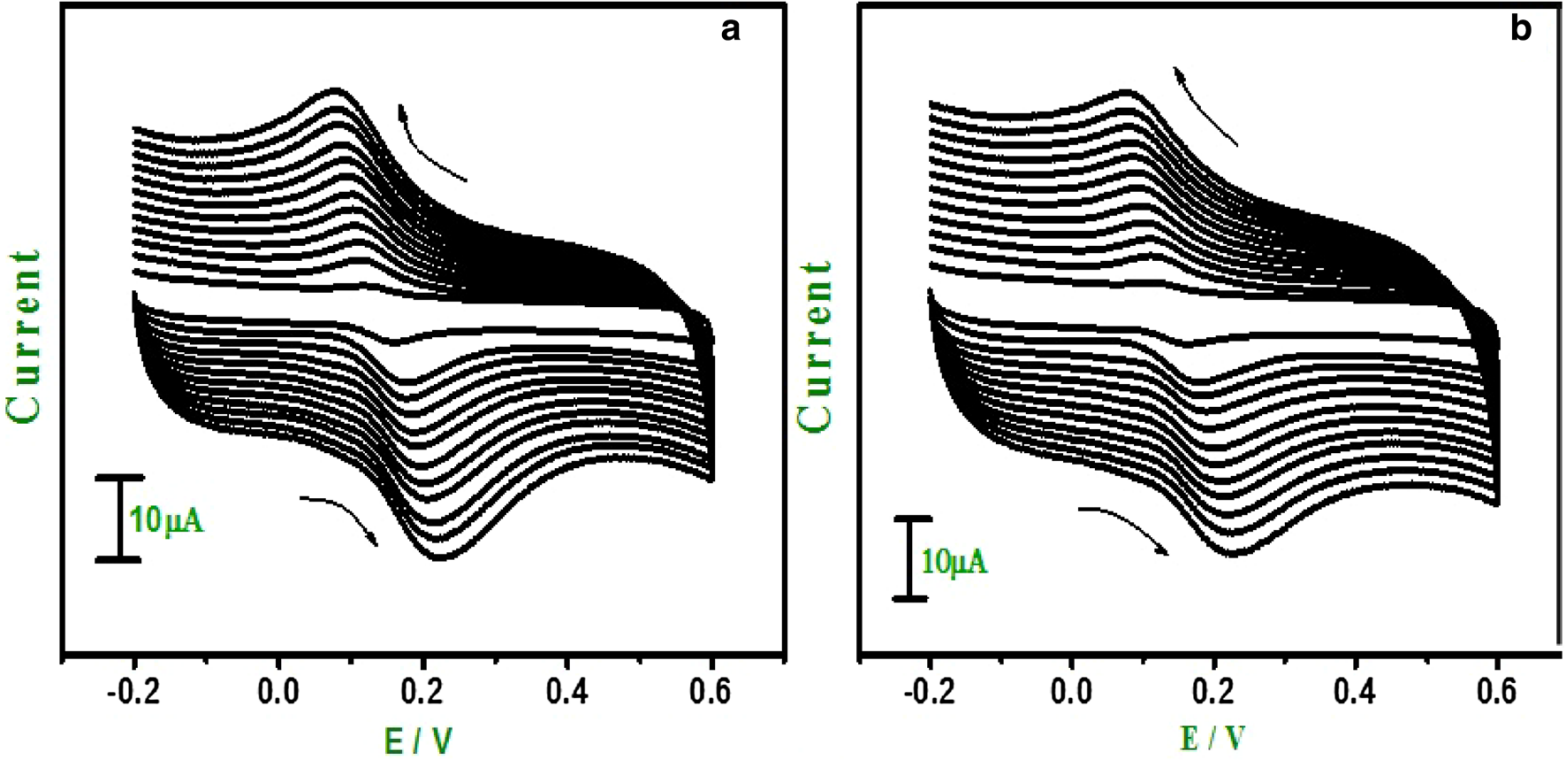
Cyclic voltammograms of 0.02 mM DA at (**a**) GO modified CPE (**b**) E-GO modified CPE in 0.2 M phosphate buffer solution at pH 7.0 with sweep rate variation (0.05–0.38 Vs^−1^).

The effect of sweep rate for DA in PBS at pH 7.0 was studied by CV at GO and E-GO modified CPE. Figure 7a,b show an increase in the redox peak currents at a sweep rate of 0.05–0.38 V s^−1^ for modified CPE prepared with GO and E-GO nanoparticles respectively. The graph obtained exhibited good linearity for the E-GO and GO modified CPE, with correlation coefficients of *r*^2^ = 0.999 and 0.999. These results indicate that the overall electrode process is controlled by a diffusion process. Using Eq. (1)^48^ heterogeneous rate constant (k^0^) was approximated.

1 $$\Delta {\text{Ep}} = 201.39\,\log( {\upupsilon/{\text{k}}^{0} }) - 301.78$$

k^0^ and peak potential difference ΔEp values are shown in Table 1. The value of k^0^ obtained at a sweep rate of 0.05 Vs^−1^ for both GO and E-GO modified CPE exhibits generously even heterogeneous rate constant, all the parameters are tabulated in Table 1.

**Table 1:** Variation of some of the parameters derived from Fig. 7, heterogeneous rate constant (k^0^) as a function of potential sweep rate.

$\upnu$(mVs^−1^)	ΔEp (mV) for GO and E-GO		k^0^ (s^−1^) for GO and E-GO	
	GO	E-GO	GO	E-GO
50	50	63	1.11	1.30
80	71	65	0.89	0.83
110	73	66	0.66	0.61
140	83	79	0.58	0.55
170	98	80	0.57	0.47
200	110	86	0.55	0.46
230	113	106	0.50	0.46
260	120	109	0.49	0.45
290	130	119	0.48	0.44
320	138	134	0.47	0.43
350	148	140	0.45	0.42
380	150	145	0.44	0.42

**Figure 8:**
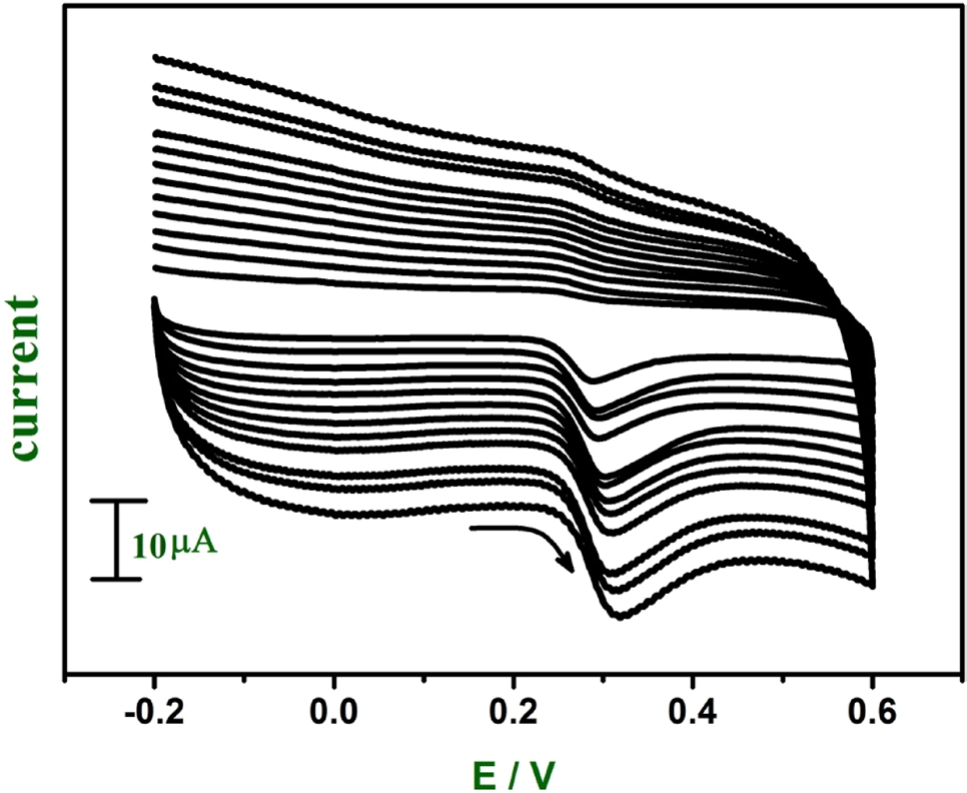
Cyclic voltammograms of 0.06 mM UA at E-GO modified CPE in 0.2 M phosphate buffer solution at pH 7.0 with sweep rate variation (0.05–0.38 Vs^−1^).

**Figure 9:**
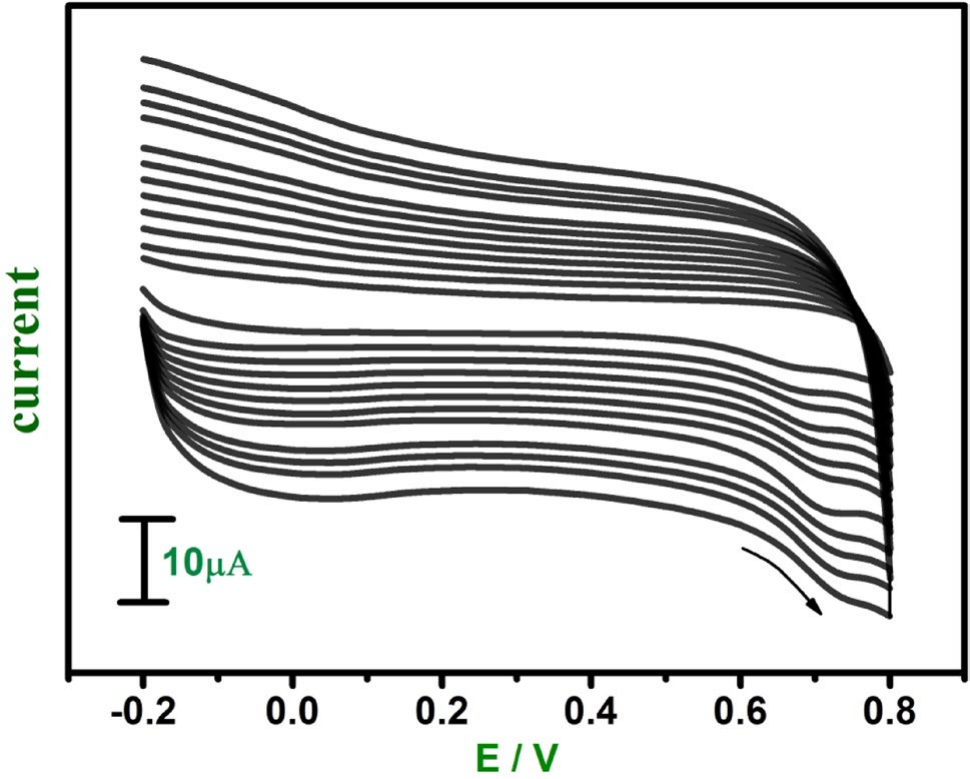
Cyclic voltammograms of 1 mM FA at E-GO modified CPE in 0.2 M phosphate buffer solution at pH 7.0 with sweep rate variation (0.05–0.38 Vs^−1^).

In the same way effect of sweep rate for UA and FA was studied by CV at E-GO modified CPE. The E-GO modified CPE showed with an increase in sweep rate 0.05–0.38 V s^−1^ the anodic peak current increases (Figs. 8 and 9). The graph obtained was good linearity between the sweep rate and anodic peak current and anodic peak current was proportional to the sweep rate for both UA and FA. The correlation coefficient for the UA was *r*^2^ = 0.991 and 0.996 for FA which indicates the electrode reaction was adsorption controlled for the modified CPE prepared with E-GO.

### Effect of concentration at E-GO modified CPE

**Figure 10:**
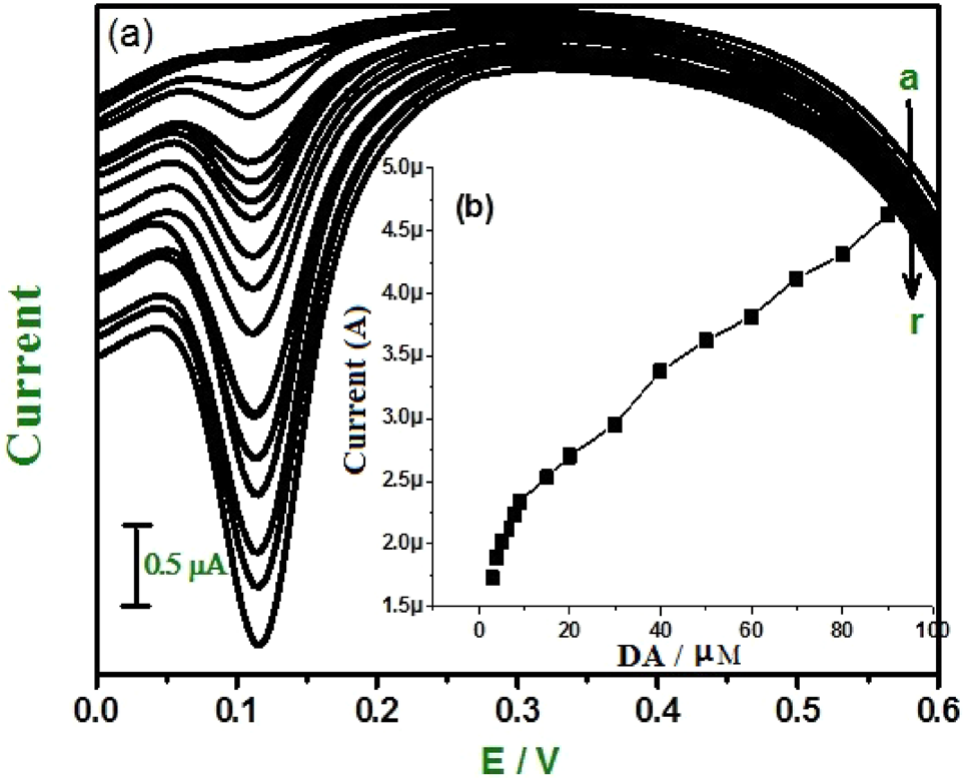
(**a**) Differential pulse voltammograms for different concentrations of DA (a-r) at E-GO modified CPE 0.2 M phosphate buffer solution. (**b**) Graph of anodic peak current vs. concentration of DA.

To obtain a much more sensitive peak current, differential pulse voltammetry (DPV) was employed. In the DPV mode background current is negligible leading to more accurate measurements. Figure 10a shows as the concentration of DA increases the peak current also increases, and the concentration of DA was varied from 0.5 to 20 µM in phosphate buffer solution at pH—7.0. Figure 10b shows the graph of Ipa versus concentration of DA with two linear relationship ranges of 0.5 × 10^−6^ M to 1.5 × 10^−6^ M and 2 × 10^−6^ M to 20 × 10^−6^ M with linear regression equation of Ipa (µA) = 0.933 C(µM/L) + 1.49 × 10^−6^ and Ipa (µA) = 0.275 C(µM/L) + 2.17 × 10^−5^, respectively. The correlation coefficient for the first linearity was 0.986, and that for the second was 0.996.

**Figure 11:**
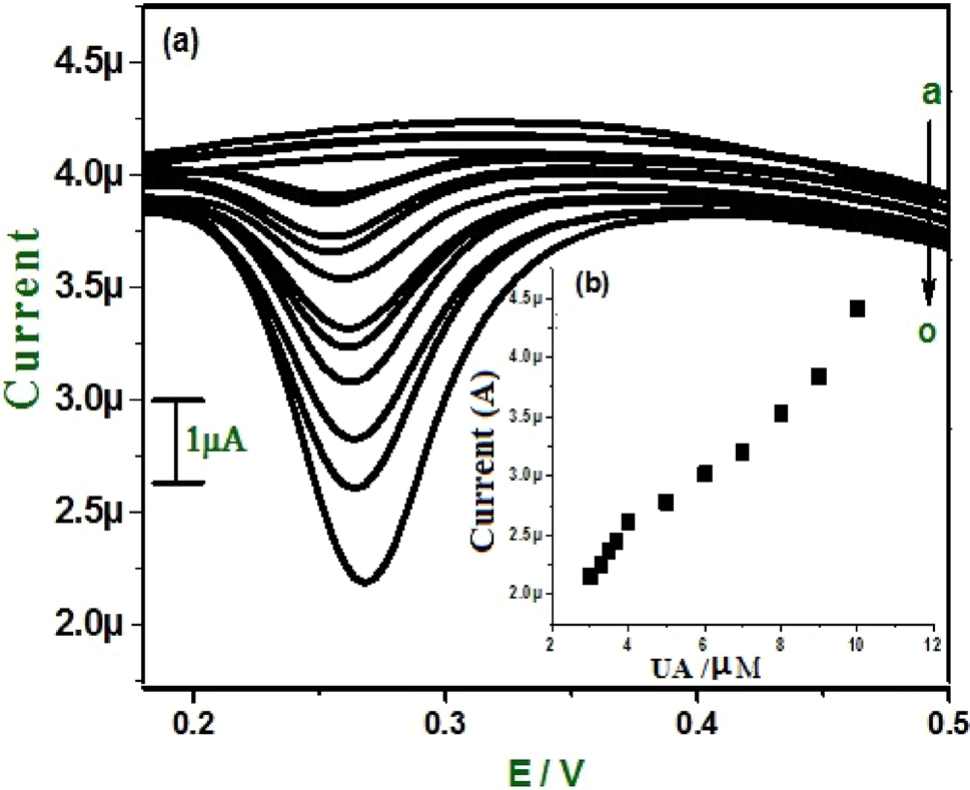
(**a**) Differential pulse voltammograms for different concentrations of UA (a-o) at E-GO modified CPE 0.2 M phosphate buffer solution. (**b**) Graph of anodic peak current vs. concentration of UA.

In the same way, the concentration of UA varied from 3 × 10^−6^ to 1 × 10^−5^ M shown in Fig. 11a. The corresponding graph of anodic peak current versus concentration of UA shows two linear relationships shown in Fig. 11b ranges of 3 × 10^−6^ M to 4 × 10^−6^ M and 5 × 10^−6^ M to 1 × 10^−5^ M with linear regression equation of Ipa (µA) = 0.465 C(µM/L) + 1.926 × 10^−6^ and Ipa (µA) = 0.312 C(µM/L) + 2.055 × 10^−5^, respectively. The correlation coefficient for the first linearity was 0.997, and that for the second was 0.981.

**Figure 12:**
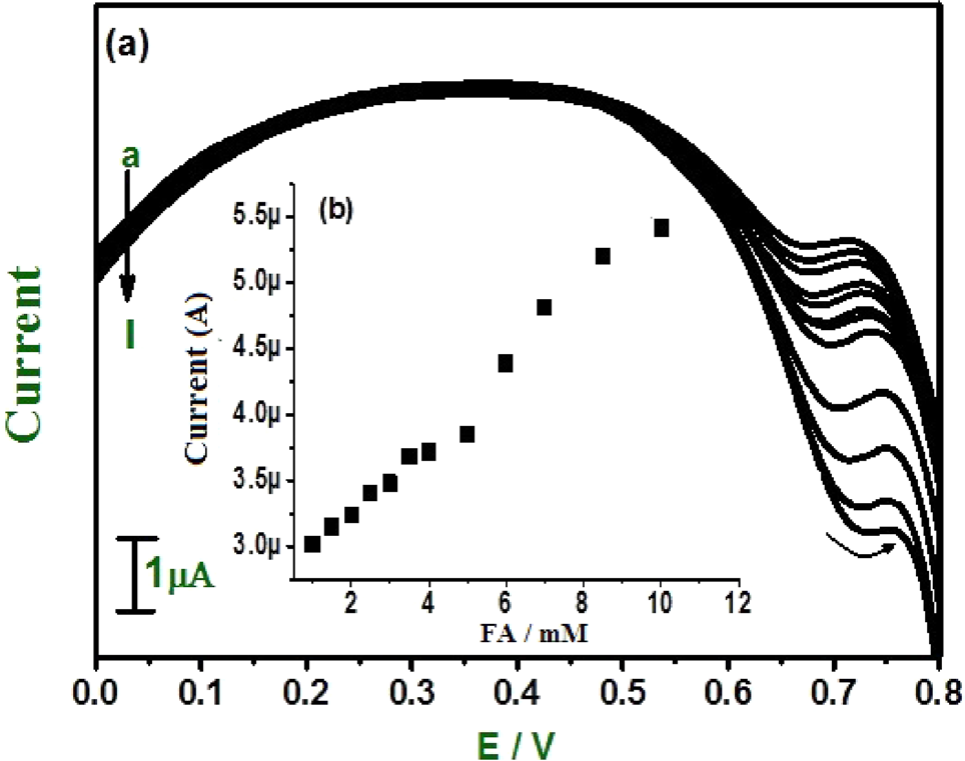
(**a**) Differential pulse voltammograms for different concentrations of FA (a–i) at E-GO modified CPE 0.2 M phosphate buffer solution. (**b**) Graph of anodic peak current vs. concentration of FA.

The concentration of FA varied from 1 × 10^−6^ to 9 × 10^−6^ M shown in Fig. 12a. Above 9 µM increasing the folic acid concentration induces a further reduction in the peak (that is anodic peak will disappear). This phenomenon was due to the presence of an amine group on one ring in the folic acid structure that could be adsorbed onto the E-GO modified CPE. These findings indicate E-GO modified CPE is suitable for the detection of folic acid at trace levels in the range of 1 × 10^−6^ M to 5 × 10^−6^ M. The corresponding graph of anodic peak current versus concentration of FA shows two linear relationships Fig. 12b ranges of 1 × 10^−6^ M to 5 × 10^−6^ M and 6 × 10^−6^ M to 9 × 10^−6^ M with linear regression equation of Ipa (µA) = 0.094 C(mM/L) + 1.401 × 10^−5^ and Ipa (µA) = 0.032 C(mM/L) + 2.136 × 10^−5^, respectively. The correlation coefficient for the first linearity was 0.997 and for the second was 0.981. The limit of detection (LOD) for DA, UA, and FA was found to be 0.031 µM, 0.21 µM, and 0.57 µM for E-GO modified CPE and was calculated^49^. The resultants LOD is comparable to previous reports, as shown in Table 2.

**Table 2:** Comparison of detection limits of DA, UA, and FA of E-GO modified CPE with other working electrodes.

Analyte	Electrode	Linear range (M)	Detection limit (M)	References
DA	SWCNT/GCE	1.0 × 10^−6^ to 1.0 × 10^−4^	7.0 × 10^−6^	^50^
	MC/GCE	5.0 × 10^−7^ to 10 × 10^−4^	2.0 × 10^−7^	^51^
	F-MWCNT/GCE	3.0 × 10^−6^ to 300 × 10^−6^	6.0 × 10^−7^	^52^
	CNT-TN/CPE	1.0 × 10^−7^ to 8.0 × 10^−5^	3.0 × 10^−8^	^53^
	E-GO/MCPE	0.5 × 10^−6^ to 1.5 × 10^−6^	3.1 × 10^−8^	Present work
UA	Helical CNT/GCE	6.7 × 10^−6^ to 6.5 × 10^−5^	1.5 × 10^−6^	^54^
	Graphene/GCE	2.0 × 10^−6^ to 1.2 × 10^−4^	6.0 × 10^−7^	^46^
	F-Graphene/CPE	1.7 × 10^−6^ to 9.0 × 10^−5^	4.5 × 10^−7^	^55^
	E-GO/MCPE	3.0 × 10^−6^ to 1.0 × 10^−5^	2.1 × 10^−7^	Present work
FA	ZrO_2_/MCPE	2.0 × 10^−5^ to 2.5 × 10^−3^	9.8 × 10^−6^	^56^
	MWCNT-PBD/CPE	30 × 10^−6^ to 800 × 10^−6^	1.0 × 10^−6^	^57^
	MWCNT/GCE	30 × 10^−6^ to 200 × 10^−6^	8.0 × 10^−7^	^52^
	E-GO/MCPE	10 × 10^−5^ to 90 × 10^−5^	5.7 × 10^−7^	Present work

### Simultaneous measurements of DA, UA, and FA

**Figure 13:**
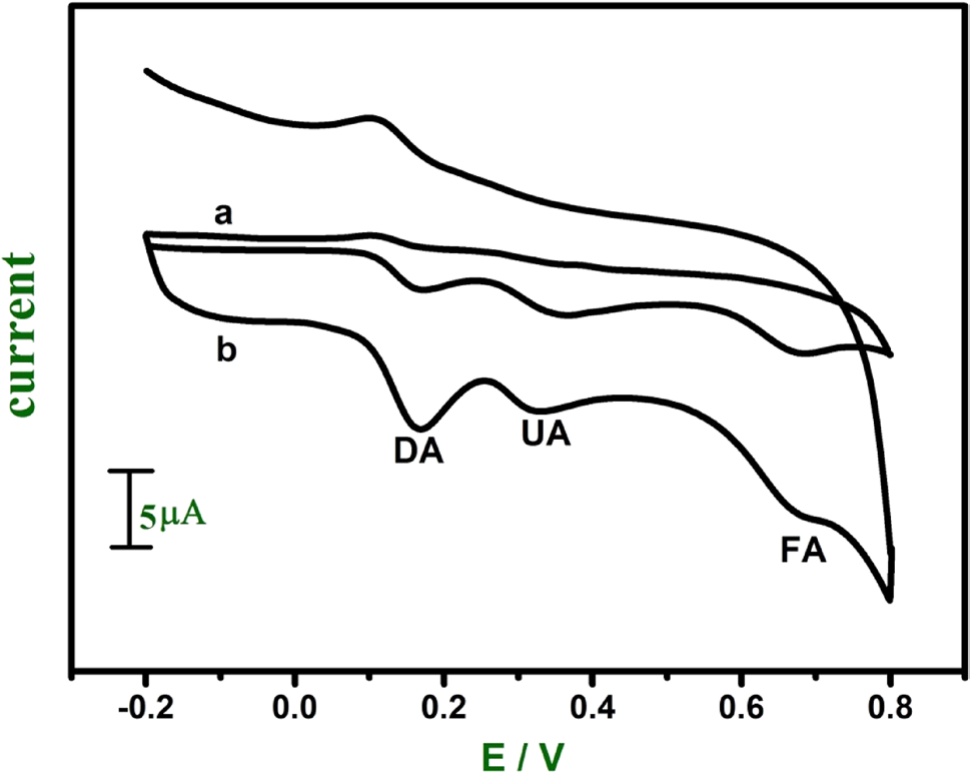
Cyclic voltammograms for a mixture of DA (0.02 mM), UA (0.06 mM), and FA (1 mM) at bare CPE (curve **a**) and E-GO modified CPE (curve **b**) in 0.2 M phosphate buffer solution of pH 7.0.

Figure 13 shows the cyclic voltammograms obtained for the electrochemical response of DA (0.02 mM), UA (0.06 mM), and FA (1 mM) at bare CPE (curve a) and E-GO modified CPE (curve b) in 0.2 M PBS. The utilization of the modifier (E-GO) shows three well-distinguished anodic peaks at potentials of 162, 323, and 674 mV for DA, UA, and FA with enhancement in the peak currents. The electrochemical peak to peak separation of DA and UA is 161 mV and for UA and FA is 351. These results suggest that identification of DA is possible in presence of UA and FA at the E-GO modified CPE.

## Conclusion

The carbon paste electrode was modified with GO and E-GO used for the detection of DA, UA, and FA by electrochemical methods. The results show the oxidation of DA, UA, and FA was catalyzed at pH 7.0 with less positive peak potentials of these analytes at the surface of the GO and E-GO modified CPE. The value of the heterogeneous rate constant obtained for the E-GO modified CPE (sweep rate 0.05 V s^−1^) exhibits a larger heterogeneous rate constant compared with other sweep rate variations. The prepared modified CPE shows a low detection limit compared with the previous works of literature and these results indicate that GO and E-GO nanoparticles are scientifically interesting and have good potential for use in sensors and other electrochemical applications.
